# Comparison of pharmacokinetics and bioavailability of bedaquiline fumarate, benzoate and maleate in dogs

**DOI:** 10.5588/ijtld.22.0326

**Published:** 2023-01-01

**Authors:** S. Jaw-Tsai, R. Barry, P. G. Pande, R. Taneja, T. Yang

**Affiliations:** 1 Sarah Jaw-Tsai Consulting Services, San Francisco, CA, USA; 2 RTI International, Research Triangle Park, NC, USA; 3 Global Alliance for TB Drug Development (TB Alliance), New York, NY, USA; 4 3DC, Deerfield Management, New York, NY, USA

**Keywords:** salts, biopharmaceutical, MDR-TB regimen

## Abstract

**BACKGROUND:**

Bedaquiline (BDQ) as a fumarate salt is indicated as part of a regimen to treat multidrug-resistant TB (MDR-TB). BDQ benzoate and maleate have been identified as promising alternatives that will encourage generic pharmaceutical houses to manufacture this drug. Our study compared the pharmacokinetics (PK) of BDQ fumarate vs. the maleate and benzoate salts in dogs.

**METHODS:**

The PK of BDQ and its active *N*-desmethyl metabolite M2 following intravenous administration of 1 mg/kg BDQ (as fumarate) and oral administration of 10 mg/kg BDQ as fumarate, benzoate, or maleate salts in suspension to fasted male beagle dogs was evaluated in a parallel-group and crossover study with *n* = 4 per group. BDQ and M2 plasma concentrations were determined up to 168 h post-dose. T-tests were conducted to compare the area under the curve, AUC_0–t_ among groups.

**RESULTS:**

Orally administered fumarate, benzoate, and maleate salts, in parallel-group design, resulted in mean BDQ AUC_0–t_ of 9,267 ± SD 10,182, 19,258 ± SD 11,803, and 15,396 ± SD 9,170 ng.h/ml, respectively; and in a crossover design of 9,267 ± SD 10,182, 17,441 ± SD 24,049, and 18,087 ± SD 19,758 ng.h/ml, respectively. *P* values were >0.05.

**CONCLUSION:**

There was no statistically significant difference in BDQ and M2 AUC_0–t_ following oral administration of fumarate, benzoate and maleate salts in dogs.

## Abstract (fr)

**CONTEXTE:**

Sous forme de fumarate, la bédaquiline (BDQ) est indiquée dans le cadre d’un schéma thérapeutique pour la TB multirésistante (MDR-TB). Le benzoate et le maléate de BDQ ont été identifiés comme alternatives prometteuses, ce qui encouragera les laboratoires pharmaceutiques de génériques à commercialiser ce médicament. Notre étude a comparé les paramètres pharmacocinétiques (PK) du fumarate de BDQ aux ceux du maléate et du benzoate de BDQ chez le chien.

**MÉTHODES:**

Les paramètres PK de la BDQ et de son métabolite actif *N*-desméthyle (M2) après administration intraveineuse de 1 mg/kg de BDQ (sous forme de fumarate) et administration orale de 10 mg/kg de BDQ sous forme de fumarate, benzoate ou maléate en suspension chez des chiens beagle mâles, à jeun, ont été évalués dans une étude croisée et en groupes parallèles avec *n* = 4 par groupe. Les concentrations plasmatiques de BDQ et M2 ont été évaluées jusqu’à 168 heures après administration de la dose. Le test de Student a été utilisé pour comparer l’aire sous la courbe (AUC_0–t_) parmi les groupes.

**RÉSULTATS:**

Dans un schéma en groupes parallèles, l’administration orale de fumarate, benzoate et maléate a été associée à une AUC_0–t_ moyenne de la BDQ de 9 267 ± SD 10 182, 19 258 ± SD 11 803, et 15 396 ± SD 9 170 ng.h/ml, respectivement ; et, dans un schéma croisé, de 9 267 ± SD 10 182, 17 441 ± SD 24 049, et 18 087 ± SD 19 758 ng.h/ml, respectivement. Les valeurs *P* étaient >0,05.

**CONCLUSION:**

Aucune différence statistiquement significative n’a été observée en termes d’AUC_0–t_ de la BDQ et du M2 après administration orale de fumarate, benzoate et maléate chez le chien.

Bedaquiline (BDQ) is indicated as part of a regimen to treat multidrug-resistant TB (MDR-TB). BDQ was introduced in 2012 as the first addition to the standard TB regimen in 50 years.^1^ The standard regimen for MDR-TB has many disadvantages such as high cost, drug interactions and less efficacy. However, addition of BDQ provided satisfactory outcome in this form of TB.^1^ Open access to this molecule is critical for keeping the cost of goods competitive, especially for developing countries where MDR-TB is prevalent. Commercially available tablets contain BDQ as fumarate salt. New salts of BDQ have been developed and characterized.^2^–^4^ These salts will encourage additional organizations to manufacture BDQ. BDQ benzoate and maleate have been identified as promising salts. This study compared the pharmacokinetics (PK) and bioavailability (BA, F) of new BDQ salts in contrast to fumarate salt in male beagle dogs, including crossover arm and parallel arm groups. The beagle dog was chosen as an animal model since dogs are frequently used in formulation development prior to clinical testing due to the lower costs and faster execution of the study. In addition, beagle dog is the most frequently used non-rodent species for PK and nonclinical safety evaluation.

## METHODS

BDQ fumarate, BDQ benzoate and BDQ maleate were prepared by Purdue University, West Lafayette, IN, USA, as described by Okezue et al.^3^ All doses and concentrations for BDQ fumarate, benzoate and maleate are stated on the basis of BDQ as free base. A correction factor of 1.215 was used for all salts. Details on materials and equipment used in this study are provided in the Supplementary Data.

### Preparation of dose formulations

The intravenous (IV) dosing solution was prepared by dissolving BDQ fumarate in 20% hydroxypropyl-β-cyclodextrin (HPβCD; pH 3, adjusted with 1N hydrochloric acid) as a 2.00 mg/ml solution, filtered through a 0.22 μm filter for IV bolus injection at 0.5 ml/kg. The oral (PO) suspension formulations were prepared by dispersing BDQ salts in 2% hydroxypropyl methylcellulose (HPMC) E15 Premium LV with 0.1% polysorbate 80 (Tween 80) as a 4.00 mg/ml suspension for dosing volume of 2.5 ml/kg. The formulation concentrations were verified using liquid chromatography tandem mass spectrometry (LC-MS/MS) by first making a 200-fold dilution in methanol, followed by a two-fold (for IV formulation) or four-fold (for oral formulations) second dilution in methanol; a 5 μl of the 400-fold (IV formulation) or 800-fold (oral formulation) diluted formulation was then added to 50 μl blank dog plasma using the procedure described for the bioanalysis of dog plasma samples. Particle size of the oral suspensions were determined using a polarized light microscope equipped with Nikon NIS-Elements BR 3.1 software (Nikon, Tokyo, Japan).

### Solubility determination

Kinetic solubility of the salts was determined in the same formulation as that used for oral administration in the animal PK studies described below. The suspensions were stirred at 300 rpm for 1 h at room temperature and then centrifuged at 14,000 r/min for 15 min. The final supernatants were diluted with the diluent acetonitrile:water (7:3, v/v) and analyzed using high-performance liquid chromatography (HPLC). Method details for the HPLC analysis of formulation concentration are provided in the Supplementary Data.

### Animal study procedures

The in-life part of the study was conducted at Shanghai BioDuro Biologics Co, Shanghai, China. Sixteen non-naïve male beagle dogs (body weight: 7.62–11.5 kg) were purchased from Beijing Marshall Biotechnology Ltd, Beijing, China. The 16 dogs were divided into four groups (*n* = 4 per group). The study design is presented in Table 1. Group 1 was assigned to receive BDQ fumarate at a dose of 1 mg/kg via IV bolus administration. The remaining three groups received BDQ salts at a dose of 10 mg/kg via oral gavage. Group 2 followed a crossover design in the order of BDQ fumarate first, followed by BDQ benzoate and BDQ maleate, with a washout period of 25 days. Groups 3 and 4 followed a parallel-group design receiving BDQ benzoate and BDQ maleate, respectively. The diet was provided throughout the in-life portion of the study except for overnight fasting prior to dosing through 4 h post-dose. Drinking water was available daily *ad libitum* to all animals.

**Table 1:** Study design and concentration and particle size of BDQ dose formulations

**A)** Study design			
Study design	Parallel↓/crossover→	Group 2 crossover	Group 2 crossover

Group 1	BDQ fumarate IV	—	—
Group 2	BDQ fumarate PO	BDQ benzoate PO	BDQ maleate PO
Group 3	BDQ benzoate PO	—	—
Group 4	BDQ maleate PO	—	—

**B)** Concentration of BDQ salts in dose formulations			

Concentration of BDQ salts in dose formulations, % nominal			

Parallel↓/crossover←	BDQ fumarate PO	BDQ benzoate PO	BDQ maleate PO

BDQ fumarate PO	96.75	98.50	114.00
BDQ benzoate PO	110.75	—	—
BDQ maleate PO	102.00	—	—

**C)** Particle size of BDQ salts in dose formulations			

Average particle size of BDQ salts in dose formulations, μm			

Parallel↓/crossover←	BDQ fumarate PO	BDQ benzoate PO	BDQ maleate PO

BDQ fumarate PO	1.53	2.59	2.50
BDQ benzoate PO	2.26	—	—
BDQ maleate PO	1.98	—	—

BDQ = bedaquiline; IV = intravenous; PO = per os (oral).

Suspensions were constantly stirred while the aliquots were withdrawn for dosing the animals. Blood samples were collected at pre-dose (0), 0.25, 0.5, 1, 2, 4, 8, 12, 24, 28, 32, 48, 54, 72, 96, 120, 144 and 168 h post-dose. For the crossover PO assessment of BDQ benzoate and maleate, an additional blood sample was collected at 24 h prior to drug administration. Plasma concentrations of BDQ and its active *N*-desmethyl metabolite, M2, were determined using LC-MS/MS.

### Bioanalysis of dog plasma samples

An LC-MS/MS method was developed for the quantitation of BDQ and M2 concentrations in dog plasma. BDQ, BDQ-M2, and internal standards (terfenadine tolbutamide and/or buspirone) were extracted from 50 μl dog plasma by protein precipitation, separated by a reversed phase HPLC column under a linear gradient with a run time of 3 min. Electrospray ionization was done using Triple Quad 5500 or 6500 (Applied Biosystems/MDS SCIEX Instruments, Foster City, CA, USA), which was operated in positive ion multiple reaction monitoring mode (BDQ and M2 *mz* transitions 555.20/58.00 and 541.20/480.10, respectively) with a dynamic range of 1.00–1,000 or 2,000 ng/ml for both BDQ and BDQ-M2. Quality control (QC) samples were prepared in dog plasma at three concentrations (2, 200, and 800 ng/ml or 2, 500, and 1,600 ng/ml) to monitor the assay performance. Details on the method parameters are provided in the Supplementary Data.

### Pharmacokinetic data analysis

The PK parameters for BDQ and BDQ-M2 were derived from the individual plasma concentration-time profiles based on non-compartmental analysis (NCA) using WinNonlin v8.0 (Pharsight, Mountain View, CA, USA). Nominal time was used for all PK calculations as actual sample collection time was within ± 10% of the designated sampling time. The half-life (*t*_1/2_) was not reportable if the coefficient of determination was less than 0.800. The mean area under plasma concentration (AUC) time curve from time zero to the time of the last quantifiable concentration (AUC_0–t_) following IV administration of fumarate salt was used to calculate oral BA of BDQ following oral administration of salts. The AUC-time curve from time zero to infinity (AUC_0–inf_) was not reportable if the t_1/2_ was not reportable or if the extrapolated AUC (AUC_extr_) represented more than 30% of the total AUC_0–inf_. In the crossover study in Group 2, a second set of PK parameters was generated using NCA after subtracting the pre-dose levels of BDQ and M2 from that post-dose.

A two-sample and two-tailed *t*-test was conducted to compare the AUC_0–t_ of BDQ and M2 for groups dosed PO following parallel-group design, i.e., Group 2 of BDQ fumarate vs. Group 3 of BDQ benzoate, and Group 2 of BDQ fumarate vs. Group 4 of BDQ maleate. A paired and two-tailed *t*-test was conducted to compare the AUC_0–t_ of BDQ and M2 for groups dosed PO following a crossover design, i.e., Group 2 of BDQ fumarate vs. Group 2 of BDQ benzoate, and Group 2 of BDQ fumarate vs. Group 2 of BDQ maleate. When *P* > 0.05, the difference between two groups is not significant.

### Ethics statement

All animals were housed in facility accredited by the Association for Assessment and Accreditation of Laboratory Animal Care and Shanghai Science and Technology Commission, Shanghai, China; the study protocol was approved by the Institutional Animal Care and Use Committee (IACUC).

## RESULTS

### Dose formulations

BDQ fumarate, BDQ benzoate and BDQ maleate were characterized by Purdue University as described by Okezue et al.^3^ Solubility of the fumarate, benzoate, and maleate salts in the oral vehicle at room temperature at 1 h was found to be 0.832 mg/ml, 0.913 mg/ml and 0.182 mg/ml, respectively.

The formulation concentrations were verified and varied between 93.5% and 114% of nominal (Table 1), which were within the acceptable ± 20% of nominal range. The nominal concentrations (2 mg/ml for IV and 4 mg/ml for PO) were used for all data analyses. Average particle size of the PO dose formulations in the parallel and crossover designs were found to be comparable (Table 1), with average range of 1.5–2.59 μm.

### Pharmacokinetics

All samples were analyzed in six batches, as described in the Supplementary Data. Following BDQ fumarate IV administration at 1 mg/kg, the plasma BDQ concentration in male dog plasma declined multi-exponentially with a terminal elimination *t*_1/2_ of 197 h (range: 101–337), with a coefficient of variation (CV) at 52% (*n* = 4). The percentage values of AUC_extr_ to AUC_0–t_ were greater than 30% from three dogs, and approximately 30% from one dog. Thus, the reportable t_1/2_ was 101 h from one dog. The average clearance (CL) from the four dogs was 1.63 ml/min/kg (CV 17.7%), or 1.59 ml/min/kg from one dog, with *t*_1/2_ of 101 h. The active *N*-desmethyl metabolite M2 mean (*n* = 4) peak concentration (C_max_) of 17.5 ng/ml was observed at 37.5 h post-dose, with a slow decline observed over a 1-week period. The M2 to BDQ AUC_0–t_ ratio was ~0.3.

Following oral administration of BDQ fumarate suspension at 10 mg/kg (Group 2), C_max_ was observed at a mean T_max_ of 3.5 h (Table 2). The mean oral BA was 14.6% (*n* = 4), with a mean *t*_1/2_ of 115 h (*n* = 2, excluded two dogs with AUC_extr_ >30% of AUC_0–t_), similar to the IV *t*_1/2_.M2 C_max_ (*n* = 4) was observed at 21 h post-dose with a mean *t*_1/2_ (*n* = 4) of 118 h, similar to that of BDQ. The M2 to BDQ AUC_0–t_ ratio was ~0.8. For Groups 3 and 4, where PK of BDQ benzoate and maleate was evaluated in a parallel-group design, the plasma BDQ concentration-time profile (Figure 1A) shows the appearance of a second peak at approximately 24 h post-dose with concentrations approaching or higher than that of the first peak, resulting in a mean T_max_ (*n* = 4) of 20 and 14.5 h, respectively. The mechanism of the second peak in the PK profile is likely due to enterohepatic circulation. The mean BDQ C_max_ (*n* = 4) following 10 mg/kg oral administration of BDQ fumarate, benzoate, and maleate suspensions to dogs in a parallel-group design was 255 (standard deviation [SD] 165), 520 (SD 115), and 535 (SD 341) ng/ml, respectively; the mean BDQ AUC_0-t_ (*n* = 4) was 9,267 (SD 10,182), 19,258 (SD 11,803), and 15,396 (SD 9,170) ng.h/ml, respectively; the mean BDQ t_1/2_ was 115 (*n* = 2), 104 (*n* = 4), 163 (*n* = 4) h, respectively; the mean oral BA (*n* = 4) was 14.6%, 23.4%, and 24.2%, respectively; the M2 to BDQ AUC_0–t_ ratio was ~1.0, 0.55, and 0.82, respectively.

**Figure:**
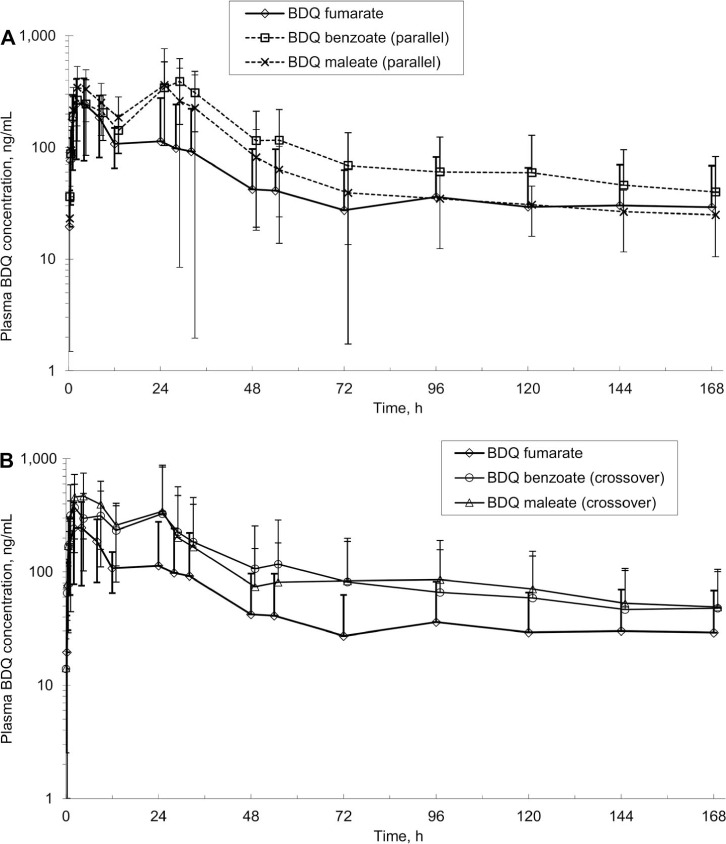
Plasma concentration-time profiles of BDQ in dogs following oral administration of 10 mg/kg BDQ in various salt forms: A) parallel-group design B) crossover design. The benzoate and maleate groups are shifted to the right for ease of reading. BDQ = bedaquiline.

**Table 2:** Non-compartmental BDQ and M2 PK following PO administration of BDQ in various salt forms to dogs

PK parameter	PO 10 mg/kg						

	Fumarate (Group 2) mean ± SD	Benzoate (Group 3) parallel mean ± SD	Maleate (Group 4) parallel mean ± SD	Benzoate (Group 2) crossover mean ± SD	Benzoate (Group 2)* crossover mean ± SD	Maleate (Group 2) crossover mean ± SD	Maleate (Group 2)* crossover mean ± SD
BDQ							
T_max_, h	3.50 ± 1.00	20.0 ± 10.8	14.5 ± 13.4	8.75 ± 10.63	8.75 ± 10.63	8.00 ± 10.71	8.00 ± 10.71
C_max_, ng/ml	255 ± 165	520 ± 115	535 ± 341	483 ± 413	469 ± 417	547 ± 409	533 ± 398
T_last_, h	168 ± 0	168 ± 0	168 ± 0	168 ± 0	133 ± 70	168 ± 0	168 ± 0
AUC_0–t_, ng.h/ml	9,267 ± 10,182	19,258 ± 11,803	15,396 ± 9,170	19,263 ± 23,594	17,441 ± 24,049	20,466 ± 21,713	18,087 ± 19,758
AUC_0–inf_, ng.h/ml	4,837 (*n* = 2)	24,357 ± 15,774	11,626 (*n* = 2)	75,218 (*n* = 1)	37,121 (*n* = 2)	42,244 (*n* = 2)	21,691 ± 23,000
t_1/2_, h	115 (*n*=2)	104 ± 27	163 ± 96	107 (*n* = 1)	53.0 (*n* = 2)	126 ± 41	87.0 ± 21.4
F, %	14.6 ± 16.0	23.4 ± 15.2	24.2 ± 14.4	30.3 ± 37.1	27.4 ± 37.8	32.2 ± 34.1	20.8 ± 22.1
M2							
T_max_, h	21.0 ± 12.8	24.0 ± 11.3	32.0 ± 0.0	15.0 ± 11.5	15.0 ± 11.4	13.0 ± 7.6	13.0 ± 7.6
C_max_, ng/ml	87.6 ± 60.8	125 ± 46	114 ± 90	132 ± 81	126 ± 82	150 ± 86	141 ± 81
T_last_, h	168 ± 0	168 ± 0	168 ± 0	168 ± 0	168 ± 0	168 ± 0	168 ± 0
AUC_0–t_, ng.h/ml	7,515 ± 5,297	10,020 ± 5,103	10,123 ± 8,523	11,717 ± 7,861	10,735 ± 8,059	14,740± 10,390	13,254 ± 9,433
AUC_0–inf_, ng.h/ml	8,363 (n=1)	15,494 ± 8,397	6,916 (*n* = 1)	NR	32,102 (*n* = 1)	27,688 (*n* = 2)	23,392 (*n* = 2)
t_1/2_, h	118 ± 43	102 ± 39	52.5 (*n* = 1)	154 (*n* = 2)	130 (*n* = 2)	89.4 ± 35.1	75.3 ± 29.5
AUC_0–t_ ratio of M2 to BDQ	1.03 ± 0.44	0.547 ± 0.112	0.819 ± 0.800	0.923 ± 0.369	1.27 ± 0.85	0.906 ± 0.241	0.949 ± 0.277

* Measurable BDQ or M2 levels at pre-dose were subtracted from those at post-dose before PK analysis.

BDQ = bedaquiline; M2 = *N*-desmethyl metabolite of BDQ; PK = pharmacokinetic; PO = per os (oral); SD = standard deviation; T_max_ = time to maximal plasma concentration; C_max_ =maximum concentration; T_last_= time at which the last quantifiable concentration was observed; AUC_0–t_= area under plasma concentration-time curve from time zero to the time of the last quantifiable concentration; AUC_0–inf_ = area under the plasma concentration-time curve from time zero to infinity; t_1/2_ = terminal half-life; F = oral bioavailability.

When PK comparison of the salts was evaluated in a crossover design with a 25-day washout (Figure 1B), the mean BDQ C_max_ following 10 mg/kg oral administration of BDQ fumarate, benzoate, and maleate suspensions to dogs was 255 (SD 165), 483 (SD 413), and 547 (SD 409) ng/ml, respectively; the mean BDQ AUC_0-t_ (*n* = 4) was 9,267 (SD 10,182), 19,263 (SD 23,594), and 20,466 (SD 21,713) ng.h/ml, respectively; the mean BDQ T_max_ (*n* = 4) were 3.50, 8.75, and 8.00 h, respectively; the mean BDQ t_1/2_ was 115 (*n* = 2), 107 (*n* = 1), 126 (*n* = 4) h, respectively; the mean oral BA was 14.6%, 30.3%, and 32.2%, respectively; the M2 to BDQ AUC_0–t_ ratio was ~1.0, 0.92, and 0.91, respectively.

In the crossover study, there were measurable pre-dose levels of BDQ (<10 ng/ml) and M2 (<10 ng/ml). The PK parameters were further calculated by subtracting the pre-dosed plasma levels of BDQ or M2 from post-dosed plasma concentrations and are also presented in Table 2. The mean BDQ C_max_ following 10 mg/kg oral administration of BDQ benzoate and maleate to dogs in Group 2 showed a decrease of 3.5% and 2.6%, respectively; the mean BDQ AUC_0–t_ decreased by 9.5% and 11.6%, respectively; the mean M2 C_max_ decreased by 4.5% and 6.0%, respectively; the M2 AUC_0–t_ decreased by 8.4% and 10.0%, respectively. The mean oral BA of BDQ fumarate, benzoate and maleate was 14.6%, 27.4%, and 20.8%, respectively; the mean BDQ t_1/2_ was 115, 53, and 87 h, respectively. The result of *t*-test on AUC_0–t_ of BDQ and M2 is given in Table 3; the *P* values were >0.05 for all comparisons conducted.

**Table 3:** T-test for AUC_0–t_ of BDQ and M2

Analyte	Groups	*P* value
BDQ (parallel design)		
Two sample equal variance, two-tailed distribution	BDQ fumarate vs. BDQ benzoate	0.247
	BDQ fumarate vs. BDQ maleate	0.405
BDQ (crossover design)		
Paired test, two-tailed distribution	BDQ fumarate vs. BDQ benzoate	0.239
	BDQ fumarate vs. BDQ benzoate*	0.335
	BDQ fumarate vs. BDQ maleate	0.154
	BDQ fumarate vs. BDQ maleate*	0.172
M2 (parallel design)		
Two sample equal variance, two-tailed distribution	BDQ fumarate vs. BDQ benzoate	0.521
	BDQ fumarate vs. BDQ maleate	0.622
M2 (crossover design)		
Paired test, two-tailed distribution	BDQ fumarate vs. BDQ benzoate	0.121
	BDQ fumarate vs. BDQ benzoate*	0.237
	BDQ fumarate vs. BDQ maleate	0.107
	BDQ fumarate vs. BDQ maleate*	0.124

* Measurable BDQ or M2 levels at pre-dose were subtracted from those at post-dose before PK analysis.

AUC_0–t_= area under plasma concentration-time curve from time zero to the time of the last quantifiable concentration; BDQ = bedaquiline; M2 = *N*-desmethyl metabolite of BDQ; PK = pharmacokinetic.

## DISCUSSION AND CONCLUSION

BDQ is a Biopharmaceutical Classification System Class II drug. Overall, the particle size of the different salts was comparable. BDQ concentrations were marginally higher for the maleate salt (117%) in the crossover arm and for the benzoate salt in the parallel arm (110%). Therefore, these factors did not bias the study results. The dosing was conducted with suspensions at 4 mg/ml. Based on the solubility data, BDQ fumarate, benzoate, and maleate salt in the oral dose formulation (2% HPMC E15 Premium LV with 0.1% Polysorbate 80) had approximately 20% of the drug in solution. Thus, the observed BA and PK parameters are mainly related to the three salt forms.

In the parallel PK study of BDQ salts in fasted male dogs, great variability of PK parameters was observed. The coefficient of variation values of AUC_0–t_ for fumarate, benzoate and maleate were 110%, 61% and 59.6%, respectively. Accordingly, a crossover study design was used in addition to a parallel-group design with the intention to minimize the variability. In the crossover design, Group 2 animals were dosed in the order of BDQ fumarate first, followed by BDQ benzoate and BDQ maleate with a washout period of 25 days. Conducting the BDQ PK study in a crossover design proves a challenge because both BDQ and M2 showed long *t*_1/2_. After a 25-day washout, all dogs in the second and third phase of the dosing had measurable concentrations of BDQ and M2 at pre-dose. The residue levels of BDQ and M2 from previous doses may have increased the exposure by approximately 10% or less by comparing the AUC_0–t_ after subtracting the pre-dose levels from that post-dose. Overall, the data from the crossover study did not suggest any reduction in variability. In this study, the plasma concentration-time profile of BDQ in dogs following oral administration revealed a second BDQ peak at around 24-h post-dose. This phenomenon has been observed with diarylquinoline compounds such as TBAJ-876 and TBAJ-587 (internal data) in preclinical and clinical PK studies. It is possible that the second peak is due to enterohepatic circulation since BDQ and its metabolites excreted in the bile (unpublished data).

The *t*-test result on AUC_0–t_ led to the conclusion that plasma exposure to BDQ and its active *N-*desmethyl metabolite M2, assessed as AUC_0–t_, following oral administration of BDQ benzoate and BDQ maleate was not statistically different from the exposure following BDQ fumarate.

In conclusion, there was no statistically significant difference in BDQ and M2 AUC_0–t_ following oral administration of fumarate, benzoate and maleate salts in dogs. Benzoate and maleate salts could offer an attractive alternative to the fumarate salt. Phase I studies will be conducted in healthy human subjects after receiving permission from the US Food and Drug Administration.
